# Use of Failure Mode and Effect Analysis Methods in Pediatric and Adolescent Hospital Care: A Scoping Review

**DOI:** 10.1097/PTS.0000000000001350

**Published:** 2025-04-15

**Authors:** Aino Färlin-Helin, Sakari Suominen, Outi Tuominen

**Affiliations:** * University of Turku; † Wellbeing Services County of Southwest Finland, Turku University Hospital, Turku, Finland

**Keywords:** patient safety, risk management, pediatrics, Failure Mode and Effect Analysis, Healthcare Failure Mode and Effect Analysis, Failure Mode and Criticality Analysis, scoping review

## Abstract

**Introduction:**

Adverse events (AEs) leading to harm to patients are prevalent across health care. However, a considerable share of AEs are preventable. Failure Mode and Effect Analysis (FMEA) has been effectively used to enhance patient safety and quality. Failure Mode and Effect Analysis (FMEA) has been effectively used to enhance patient safety and quality. This scoping review aims to provide an overview of the studies reporting the use of FMEA, failure mode and criticality analysis (FMECA), and health care Failure Mode and Effect Analysis (HFMEA) in pediatric and adolescent hospital care.

**Methods:**

We conducted a systematic search of Web of Science, Scopus, Embase, Cochrane, CINAHL, and PubMed for relevant literature published since 1999. Papers were analyzed based on the FMEA process steps.

**Results:**

Eighteen papers were included in the review, assessing 21 processes, primarily involving drug prescribing, dispensing, and administration. Participants in the risk assessment came from various occupational groups. Risk priority numbers varied based on severity, occurrence, and detection. A total of 220 high-risk risk priority numbers were identified. Improvement actions had not been systematically reported.

**Conclusions:**

FMEA, FMECA, and HFMEA were successfully used to ensure patient safety in pediatric and adolescent hospital care. These methods can be used to effectively identify possible failures in healthcare processes and in quality improvement and risk reduction. They also enable prioritizing the targets of improvement actions. In addition, the use of risk analysis methods may result in increased awareness of potential safety risks among the workers who have participated in risk assessment.

Pediatric patient harm differs from that experienced by adults. Reasons for the unique domains of patient safety problems are related to the physical characteristics of children, their developmental stage, and their role as minors.^1^ The most typical adverse events (AEs) among child and adolescent patients are associated with surgical events,^2^ medication and fluid management,^3–5^ and health care–associated infections.^2,3^ Efforts to reduce AEs among pediatric patients have been successful, but overall rates are still high.

In 2018, Stockwell et al^6^ reported 10.9 adverse events (AEs) per 100 pediatric admissions across 16 American hospitals from 2007 to 2012. Half of these AEs are preventable.^6^ These include medication, communication, patient identification errors, and issues with equipment and monitoring patients.^7^ The types of preventable harm are similar to the types of common AEs. The consequences of AEs can be serious and can lead to prolonged hospital stays or patient death.^8^ AEs also incur significant unnecessary costs;^9^ ~606 billion dollars is spent annually within OECD countries on treating avoidable AEs.^10^ It is estimated that eliminating harm could improve global economic growth by over 0.7% a year.^10^

Failure Mode and Effect Analysis (FMEA) is a proactive risk management method.^11^ It can be used in preprocess and postprocess alterations, designing a new product or service, before implementation of a new process,^12,13^ and in reducing health care risks and improving patient safety.^12–15^ FMEA is a systematic process, originally including 5 steps (Fig. 1). When used for criticality analysis, the process can be referred to as failure mode, effect and criticality analysis (FMECA). The modified version for use in the health care environment is called health care Failure Mode and Effect Analysis (HFMEA). FMECA and HFMEA follow the same procedures as FMEA, but they have specific functions.^17^ Besides improving care processes, HFMEA can reinforce a culture of continuous quality improvement.^11^

**FIGURE 1:**
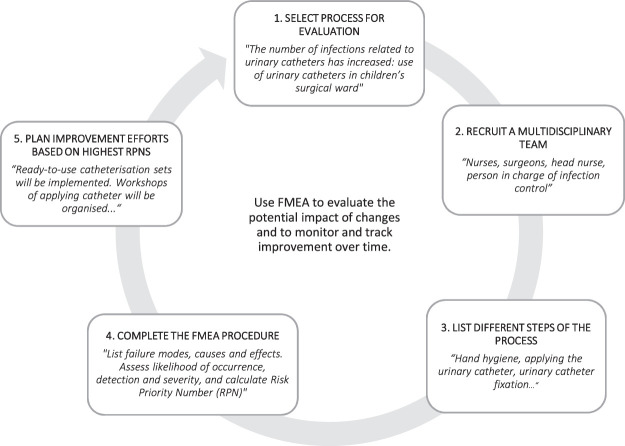
The FMEA process. Data from Institute for Health care Improvement: QI essentials toolkit: Failure mode and effects analysis (FMEA).^16^

FMEA is a practical tool for enhancing health care quality and reducing errors.^12^ It has been widely used to improve various health care processes, such as medication safety.^18^ It has been applied in the design and implementation processes of analgesia, sedation, and paralysis order set in pediatric intensive care units (PICUs),^19^ in the administration of chemotherapy to hospitalized children with cancer,^20^ and in improving the workflow and quality of hospital pharmacy services.^21^ It also has been utilized in risk assessment of organ transplant operations^22^ and in improving the quality of hospital sterilization processes.^23^

FMECA has been used to optimize chemotherapy medication leftover management,^24^ evaluating acute stroke diagnostic process,^25^ and in neonatal parenteral nutrition production process.^26^ HFMEA has been used in the administration of chemotherapy in a pediatric oncology ward^27^ and in preventing parenteral nutrition medication errors with newborns.^28,29^

Although FMEA, FMECA, and HFMEA have been applied in pediatric and adolescent hospital settings, systematic research into the types of processes they address and the outcomes achieved remains limited. This scoping review aims to provide a comprehensive picture of FMEA’s application in children’s and adolescent’s hospital care by systematically identifying and presenting existing research outcomes.

## METHODS

The aim of this scoping review is to provide an overview of studies reporting the use of FMEA, FMECA, and HFMEA in pediatric and adolescent hospital care. The secondary aim is to identify literature gaps that could inform future research or develop clinical practice.

The methodological approach for this scoping review followed the framework of Levac et al,^30^ which complements the original framework of Arksey and O’Malle.^31^ The framework consists of 6 steps: identifying the research question, identifying the relevant studies, study selection, presenting the data, collating the results and consultation (optional). This scoping review is reported in accordance with the Preferred Reporting Items for Systematic Review and Meta-Analyses Extension for Scoping Reviews (PRISMA-ScR).^32^

### Identifying the Relevant Studies, Search Strategy, and Data Sources

Six electronic databases were searched for relevant articles: Web of Science, Scopus, Embase, Cochrane, CINAHL, and PubMed. The selected databases were deemed most likely to contain studies relating to patient safety. Second, manual techniques were used to review the reference lists of the included studies. Third, searches were carried out via Google Advanced Search. The searches were conducted on March 19, 2024.

The following Medical Subject Headings (MeSH) and search terms were used: health care failure mode and effect analysis, HFMEA, FMECA and hospital. Results were limited to the English language. Searches were performed on data from 1999 to March 2024. An electronic reference manager (Zotero) was used to store the search results by databases and for identification of duplicated articles. Search terms and criteria are reported in Table 1.

**TABLE 1:** Search Terms and Criteria

Database	Search terms entered	Date of search	Results
PubMed	(“Health care Failure Mode and Effect Analysis”[Mesh] OR “HFMEA” OR “FMECA” OR “failure mode, effects and criticality analysis” OR “failure mode effects and criticality analysis” OR “FMEA” OR “failure mode and effect analysis”) AND (“hospital” OR “Hospitals”[Mesh])	19/3/24 Results were limited to 1999 present day (03/24)	370
CINAHL	(“Health care Failure Mode and Effect Analysis” OR “HFMEA”) OR (“failure mode, effects and criticality analysis” OR “FMECA” OR “failure mode effects and criticality analysis”) OR (“failure mode and effect analysis” OR “FMEA”) AND (“hospital”)	19/3/24 Results were limited to 1999 present day (03/24)	137
Web of Science	“Health care Failure Mode and Effect Analysis” OR “HFMEA” (topic) OR “FMECA” OR “ failure mode effects and criticality analysis” OR “failure mode, effects and criticality analysis” (topic) OR “FMEA” OR “failure mode and effect analysis” “AND” “hospital”	19/3/24 Results were limited to 1999 present day (03/24)	1190
Cochrane	“Health care Failure Mode and Effect Analysis” OR “HFMEA” (title, abstract, keyword) OR “FMECA” OR “failure mode effects and criticality analysis” OR “failure mode, effects and criticality analysis” (t/a/kw) OR “FMEA” OR “failure mode and effect analysis” (t/a/kw) AND “hospital”	19/3/24 Results were limited to 1999 present day (03/24)	12
Scopus	Article title/abstract/keyword “Health care Failure Mode and Effect Analysis” OR “HFMEA” OR “FMECA” OR “failure mode effects and criticality analysis” OR “failure mode, effects and criticality analysis” OR “FMEA” OR “failure mode and effect analysis” AND “hospital”	19/3/24 Results were limited to 1999 present day (03/24)	521
Embase	(“Health care Failure Mode and Effect Analysis” OR “HFMEA” OR ‘health care failure mode and effect analysis’/exp) OR (“FMECA” OR “failure mode effects and criticality analysis” OR “failure mode, effects and criticality analysis”) OR (“FMEA” OR “failure mode and effect analysis” OR ‘failure mode and effects analysis’/exp”) AND (“hospital” OR ‘hospital’/exp”)	19/3/24 Results were limited to 1999 present day (03/24)	834
	All searches were limited to English language.		2968
Google Advanced Search	“HFMEA” or “health care failure mode and effect analysis” or “FMECA” OR “failure mode, effects and criticality analysis” OR “failure mode effects and criticality analysis” OR “FMEA” OR “failure mode and effect analysis” AND “hospital”	19/3/24 Results were limited to 1999 present day (03/24)	147

### Study Selection

Inclusion criteria were that studies reported the use of a Failure Mode and Effect Analysis for risk assessment (FMEA, FMECA, or HFMEA) and the outcome of risk assessment (risk priority number, the hazard score or risk classification). The setting was a children’s hospital, and children’s hospital staff participated in the assessment.

After duplicates identified by Zotero were removed, titles and abstracts were independently screened by 2 authors (A.F.-H. and O.T.). Screening agreements were tested with the first database results after 20 and 40 records to ensure a common understanding. After screening titles and abstracts of all databases, the authors (A.F.-H. and O.T.) discussed the outcomes. Studies that did not report outcomes following the FMEA protocol were excluded. After screening full texts (n = 98), 16 studies were included. In addition, one study was obtained with a manual search using reference lists, and one was found through Google Advanced Search. Altogether, 18 studies were included in the final review.

### Data Analysis

First, the data were charted, and the synthesized results were categorized based on key characteristics: authors and year of publication, country (where the study was conducted), study design, study environment (type of unit), participants, the aim of the study, and risk analysis method. Results were discussed and reviewed among the authors. Second, the charted data and the synthesized results of the Failure Mode and Effect Analysis (eg, calculated risk priory number RPN or method; FMEA, FMECA, and HFMEA) were reviewed at intervals, including meetings and discussions between 2 authors (A.F.-H. and O.T.). In cases of any uncertainties, a third author (S.S.) was consulted. The framework for synthesizing the results followed the FMEA risk assessment method.

## RESULTS

The results are presented according to the FMEA process: select a process for evaluation, recruit a multidisciplinary team, list the steps of the process, complete the FMEA procedure and plan improvement efforts based on the highest risk priority numbers (RPNs).

### Overview of Included Studies

**FIGURE 2:**
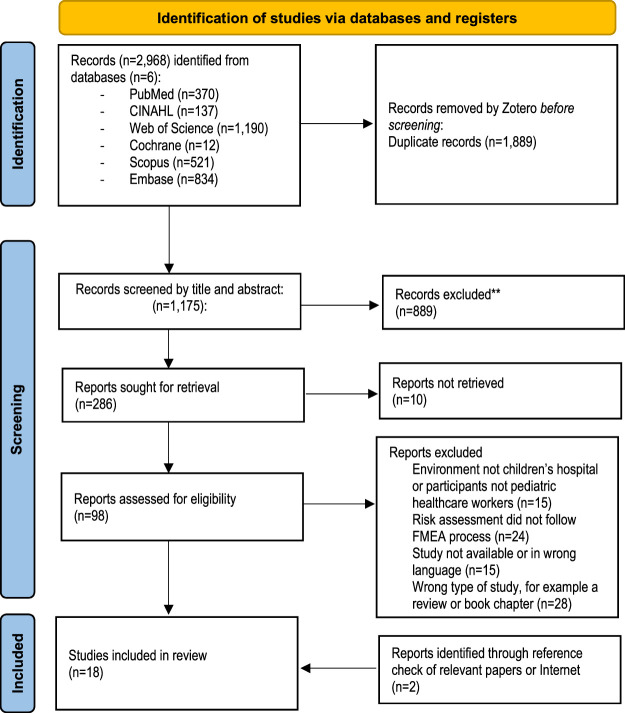
Study selection based on the Preferred Reporting Items for Systematic Reviews and Meta-Analysis flow diagram, available online (www.prisma-statement.org).

A total of 3064 articles were retrieved from databases. Of those, 18 studies were included in the review (Fig. 2). The selected studies were published between 2004 and 2022. FMEA had been used in 14 studies,^33–46^ and FMECA and HFMEA^47,48^ in 2. In one study, both FMEA and FMECA were used.^35^ In all of the studies, the RPN (or hazard score or risk classification) was used and reported. Six studies were conducted in neonatal intensive care unit (NICU),^33,37,43,45,48,49^ 3 in pediatric intensive care unit (PICU),^34,36,39,40^ 2 in a pediatric emergency department,^35,42^ 1 in a surgery department^47^ and one in an operating room.^41^ In 6 studies, the environment included 2 or more units,^45,50^ the whole department^38,42,47^ or the entire hospital.^44,46^

All included studies had applied methodology accompanied by quantitative data analysis. Data had been collected mainly using FMEA. Some studies have reported the use of an electronic worksheet.^38,49^ Observations,^33,35,37,41,44,47^ clinical audits,^40^,^41^ focus group interviews,^33,42,47^ interviews of the parents,^45^ reviewing incident reports,^36,42^ and benchmarking^43^ had been used for data collecting.

### Select Process for Evaluation

Altogether, 21 different processes were assessed for risks. The majority (n = 11) of processes consisted of prescribing, dispensing, and administrating drugs.^33,34,36–41,43,44,50^ Implementation and use of smart pumps was assessed.^40^ Two processes were about hygiene, more precisely, infection control and central line–associated bloodstream infections.^33,49^ Risks in pediatric surgery^47^ and in emergency department care processes^42^ were evaluated. The rest of the processes were about the use of medical equipment,^33^ laboratory tests,^33^ infant misidentification, and abduction,^45^ human milk processes in the NICU,^48^ and simulation-based system testing process.^46^

### Recruit a Multidisciplinary Team

Employees taking part in the risk assessments represented various occupational groups. The team leaders were either someone in charge of risk management^44,47^ or a process improvement engineer.^44^ In some studies, the team had been assisted by an external facilitator trained in FMEA.^36,38,43^ Members of the FMEA assessment teams had been selected based on their experience,^33^ level of involvement in the respective processes,^33,34^ clinical experience^38^ and expertise,^50^ for example, 2 years in the intensive care unit,^43^ interests,^50^ availability for the project^43,50^ and profession,^46,48^ for example, nurse or medical doctor (Table 2).

**TABLE 2:** Characteristics of Included Studies

Author	Country	Study design	Study environment	Participants	Aim	Risk analysis method
Alimohammadzadeh et al^33^	Iran	Cross-sectional study	NICU	Neonatologist, pediatrician, NICU nurse, NICU head nurse, the infection control supervisor, laboratory supervisor, head of quality improvement unit, expert in medical equipment. (n = 8)	To identify and assess common medical errors and their effects.	FMEA
Apkon et al^34^	USA	Cross-sectional study	PICU	Pediatric intensivist, pharmacist, nurse specialist, hospital epidemiologist, quality management administrator. (n = NA)	To develop a set of standard processes for delivering continuous drug infusions and examine the impact of process changes.	FMEA
Bagnasco et al^35^	Italy	Prospective cohort study	Pediatric emergency department	Physicians and nurses working in the pediatric ED. (n = 43)	To identify effective corrective measures to ensure patient safety.	FMEA, FMECA
Chandonnet et al^49^	USA	Prospective cohort study	NICU	Clinicians for nursing, medicine, surgery, pharmacy, infection prevention and quality improvement. (n = NA)	To analyze reasons for increase in the number of central line–associated blood stream infections and implement corrective actions.	HFMEA
Daverio et al^36^	Italy	Prospective cohort study	PICU	Medical doctors and nurses from neonatal ICU, PICU, pediatric acute care unit, pediatric onco-hematology unit, and general pediatric unit, external facilitator trained on the FMEA. (n = NA)	To describe the trend of incident reports and evaluate the effect of FMEA application.	FMEA
Kunac et al^37^	New Zealand	Cross-sectional study	NICU	Management representatives (organizational level, pharmacy, 2 neonatal unit), front-line clinical staff representatives (2 medical, nursing, pharmacy). (n = 8)	To identify and prioritize potential failures in the NICU medication use process.	FMEA
Lago et al^38^	Italy	Cross-sectional study	Pediatric department	Doctors, residents, nurses, patient safety and risk management experts, quality management experts, pharmacist, representative of service for health professions administrative officer from different pediatric units, and external facilitator with training in FMEA. (n = 40)	To analyze the drug-delivery process, to identify possible causes of failures and their potential effects and plan changes in practices.	FMEA
Malfara et al^39^	Brazil	Prospective observational study	PICU	Four medical doctors, 5 pharmacists, 5 nurses, 5 nursing assistants/technicians, and an administrative officer. (n = 20)	To evaluate the number and type of clinical pharmacist interventions and to determine cost savings associated with them.	FMEA
Manrique-Rodriguez et al^40^	Spain	Cross-sectional study	PICU	Two intensive care pediatricians, 2 clinical pharmacists, PICU nurse manager. (n = 5)	To identify possible actions for improvement and to assess the effects that those improvements could have on the risk points detected. To assess the risks of the use of smart infusion pumps before and after implementation.	FMEA
Martin et al^41^	USA	Prospective cohort study	Operating room	Anesthesiologists, pharmacists, nurses, process improvement consultant. (n = NA)	To identify risks in intraoperative medication handling process, implement countermeasures and collect medication error data.	FMEA
Molavi-Taleghani et al^47^	Iran	Cross-sectional study	Pediatric surgery department	Person in charge of risk management, health care manager, supervisor, professor in the field of pediatric surgery, 2 nurses, physician, laboratory expert, and health information expert. (n = 9)	To examine the risk assessment of processes in pediatric surgery.	HFMEA
Mora-Capin et al^42^	Spain	Prospective cohort study	Pediatric emergency department	Physicians, nursing staff, including safety representatives, quality assurance representatives, and consultants of the risk management operations unit. (n = NA)	To identify and manage the risks through the development of a risk map, to assess the impact of the implemented improvement actions.	FMEA
Najar et al^43^	Iran	Cross-sectional study	NICU	Two nurses, physician, FMEA expert, consultant in charge of risk management, person in charge of hospital quality improvement. (n = 6)	To identify and analyze the potential errors in drug prescription and administration processes.	FMEA
Nguyen et al^50^	Canada	Cross-sectional study	Two pediatric units	Head pharmacist, 2 pharmacists, 2 pharmacy residents, pharmacy research assistant, physician nephrologist, 2 nurses, hospital risk manager. (n = 10)	To evaluate the causes of adverse drug events during the nurse medication administration process.	FMECA
Pino et al^44^	USA	Cross-sectional study	Academic pediatric hospital	Two process improvement engineers, pharmacist clinical safety leader, representatives from different pediatric units. (n = NA)	To identify safety risks of unfractionated heparin use and to develop and implement countermeasures to improve safety.	FMEA
Webster et al^45^	USA	Cross-sectional study	Center for women and infants, labor and delivery unit, mother and baby unit, triage rooms, NICU.	Nurses, pediatrics, security, process improvement. (n = NA)	To proactively identify and eliminate or reduce the risk of infant misidentification or abduction.	FMEA
Williams et al^46^	USA	Cross-sectional study	Children’s hospital	Clinical and administrative stakeholders from trauma/surgery, emergency medicine, OR, nursing, respiratory therapy departments. (n = NA)	To describe simulation-based clinical system testing process, evaluate its impact on patient safety, and estimate financial costs and benefits.	FMEA
Zhang et al^48^	USA	Cross-sectional study	NICU	Staff nurses, nurse managers, clinical nurse specialist, nursing education specialist, lactation consultant, physician, nurse administrator, system engineer. (n = NA)	To evaluate the levels of risk of the human milk processes, identify actions to reduce process risk and quantify the impact of process changes.	HFMEA

ED indicates emergency department; NICU, neonatal intensive care unit; OR, operating room; PICU, pediatric intensive care unit.

### List Different Steps of the Process and Complete the FMEA Procedure

Steps of the processes, for example a drug dose prepared with inadequate technique^50^ or discharge management,^42^ and the number of failure modes varied among the studies (Table 3). According to the FMEA process, causes and effects for failure modes were considered.

**TABLE 3:** Processes and Risk Priority Numbers (RPNs)

Name of the risk assessment method	Process to be analyzed	Scale for severity (S), occurrence (O), detection (D)	Failure modes	Limit for high-risk RPN	Number of high-risk RPNs	Examples of FMs with highest risk priority number (RPN)	Study
FMEA	Drug administration, infection control, use of medical equipment, laboratory tests	S = 1-5 O = 1-10 D = 1-10	NA	>65	27	Mistakes in drug calculation (115) Incomplete washing and disinfecting hands (127) Defects in the quality of using medical equipment (104)	Alimohammadzadeh et al^33^
FMEA	Process of the continuous drug infusions	S, O, D= 1-10	NA	>150	3	Programming the pump (269) Prepare the drug infusion (314) Calculate the dose (234)	Apkon et al^34^
FMEA	Medication process	NA	NA	NA	NA	Miscalculation (60) Wrong dilution (60) Wrong rate (60)	Daverio et al^36^
FMEA	NICU medication process	NA	72	>212.5	30	Lack of medication safety training (273) Incorrect dose administration (265) Prepare medication (252)	Kunac and Reith^37^
FMEA	Drug prescribing and administering	S, O, D = 1-5	702	>48	37	Prescription (NA) Preparation of the drug (NA) Calculation of the dose (NA)	Lago et al^38^
FMEA	Clinical pharmasist interventions	NA	75	NA	NA	Lack of validation of the medical prescription by the pharmacist before dispensing (640) Lack of pharmacist participation in multidisciplinary discussions (576) Lack of monitoring of serum levels of drugs by the clinical pharmacist (576)	Malfara et al^39^
FMEA	Implementation of smart pumps	S, O, D = 1-10	20	>63	11	Failure to comply with protocols regarding standard concentrations administration times in intermittent infusions (210) Slow download speed (189) Slow data upload/update (189)	Manrique-Rodriguez et al^40^
FMEA	Intraoperative medication handling process	S = 0-5 O, D = 1-5	69	>30	15	Inadequate knowledge and information (60) Dilution error (60) Wrong drug (60)	Martin et al^41^
FMEA	Pediatric emergency department process	S, O, D = 1-5	106	>75	58	Treatment (NA) Diagnostic test (NA) Discharge management (NA)	Mora-Capin et al^42^
FMEA	NICU drug prescription and administration	S, O, D = 1-5	68	>100	5	Errors in prescription method (60) Incomplete comment in physician order (40) Allergic reaction of the patient to the prescribed drug (40)	Najar et al^43^
FMEA	Unfractioned heparin use	S = 1,3,5-10 L*,D = 1,3,5,8,10	233	> 500	45	Mathematical errors (NA) Multiple practices are acceptable/requirements not known (NA) UFH can be given at any (incorrect) time (NA)	Pino et al^44^
FMEA	Infant misidentification or abduction	NA	28	> 12	12	Patients are escorted from the emergency department to the psychiatric unit through the circumcision area where infants may be lying (48) Safety and communication drills are only once a year (48) Aggressive individuals allowed to enter the labor and delivery ward due to propped doors, no posted security, or others holding the door open (48)	Webster et al^45^
FMEA	Managing change-associated risks in pediatric trauma center	S = 1-4 P† = 1-4	49	> 8	38	Equipment/supply availability (16) Staffing (16) System issues (issues related to processes or procedures that do not work as well as anticipated in the clinical setting (16)	Williams et al^46^
FMEA	Human milk process	NA	50	NA	32	NA	Zhang et al^48^
FMEA, FMECA	Communication in emergency department	S, O = 1-10 D = 10-1	22	>100	NA	Filling in chart for transfer/discharge. Chart not shared (366) Drug administration (384) Report on admission on accident (512)	Bagnasco et al^35^
FMECA	Nurse medication administration process	Frequency (occurrence) = 1-9 Detection 0%-100% Severity 1-9	53	NA	NA	Drug dose prepared with inadequate technique (551) Drug dose inadequately identified (551) Absence of advice to the patient (551)	Nguyen et al^50^
HFMEA	Central line–associated bloodstream infections	S, O = 1-5 D 1-4	67	>18	13	Contamination (36) Suboptimal environment of care (24) Inadvertent disconnection dislodgement (27)	Chandonnet et al^49^
HFMEA	Pediatric surgery process	NA	218	>8	8	Request for unnecessary tests by the physician (9) Error in entering the test results (9) The patient’s delay in entering the ward for making the organizations (9)	Molavi-Taleghani et al^47^
					220		

* L = likelihood of occurrence.

^†^Probability of occurrence.

Values for severity (S), detection (D), and occurrence (O) had been estimated. Risk priority numbers had been calculated (S x D x O), and the scale for each was from 1 to 10.^16^ In some studies, an adapted version of the RPN scale had been used.^33,38,41–44,46,50^ In one FMECA study, detection had been reported in percent.^50^ In 6 studies, the scale had not been reported (Table 3).^36,37,39,45,47,48^

### Plan Improvement Efforts

Improvement actions or recommendations for improvement of high-risk events (n = 220) were presented in 18 studies. These 443 actions varied according to the purpose of the study. For example, studies that aimed at improving medication safety included double-checking for every medication or fluid prescription process,^36,38,41^ introduction of the use of standard doses or dilutions^34,36,38^ or launching systematic staff training (See Table 4).^43,50^

**TABLE 4:** Improvement Actions and Evaluation

Study	Process for analyzation	Examples of FMs with highest Risk Priority Number (RPN)	Improvement actions reported (n*) with examples	Method for evaluation of improvement actions	RPN recalculated
Alimohammadzadeh et al^33^	Drug administration, infection control, use of medical equipment, laboratory tests	Mistakes in drug calculation (115) Incomplete washing and disinfecting hands (127) Defects in the quality of using medical equipment (104)	NA	NA	NA
Apkon et al^34^	Process of the continuous drug infusions	Programming the pump (269) Preparation of the drug infusion (314) Calculation of the dose (234)	Yes (n = 7) Standard formulations required for infusion Database of approved formulations Development of calculators for various computer platforms Redesign of CPOE (computerized physician order entry) screens Extended shelf life from 24 to 72 hours	Survey	The original process had RPNs > 225. The revised process had no elements with RPNs > 100
Daverio et al^36^	Medication process	Miscalculation (60) Wrong dilution (60) Wrong rate (60)	Yes (n = 3) Double checking and signing for each medicine prescription Using the same references for standard doses and dilutions The use of a dedicated and isolated area when prescribing medicines to reduce distractions	Incident reports. Implementation of FMEA led to an increase in incident reporting and reduction in the severity or errors reported.	NA
Kunac and Reith^37^	NICU medication process	Lack of medication safety training (273) Incorrect dose administration (265) Preparation of the medication (252)	Yes (n = 3) Medication safety training Use of oral syringes Safe storage of medications	Monthly audits	NA
Lago et al^38^	Drug prescribing and administering	Prescription (NA) Preparation of the drug (NA) Calculation of the dose (NA)	Yes (n = 27) Quiet place for preparing prescriptions without distraction; tables for standard doses and dilutions Daily discussion of clinical situation and ongoing therapy between resident and attending physicians. Nurse double-checks and double-signs for preparation; nurse signs for drug administration Written instructions for parents involved in drug administration Check vital signs and site of infusion for certain drugs	Clinical audits 3 and 6 months after completing the FMEA process	After the implementation of corrective actions, none of the steps in the revised drug administration process had RPNs > 32. The reduction in the RPNs for the higher risks was around 60% at almost all units, and 23 of 37 higher risk failure modes identified with FMEA.
Malfara et al^39^	Clinical pharmasist interventions	Lack of validation of the medical prescription by the pharmacist before dispensing (640) Lack of pharmacist participation in multidisciplinary discussions (576) Lack of monitoring of serum levels of drugs by the clinical pharmacist (576)	Yes (n = 197) Main type was related to allergies, drug interactions and therapeutic monitoring, drug selection, drug dose and frequency, and drug administration	Assessment of economic benefits	NA
Manrique-Rodriguez et al^40^	Implementation of smart pumps	Failure to comply with protocols regarding standard concentrations and administration times in intermittent infusions (210) Slow download speed (189) Slow data upload/update (189)	Yes (n = 13) Assess new drugs for possible inclusion Remove unnecessary lines Double check data entered Contact manufacturer, schedule updates in advance, staff collaboration, identify pumps that have not been updated, radio frequency systems Use smart towers, port multipliers or WiFi antennas	Ongoing audits	18 months after the smart pump technology was introduced, it was found that most of the risk points identified had disappeared. However, no improvements were made to the data update and download process after the initial stages of the study.
Martin et al^41^	Intraoperative medication handling process	Inadequate knowledge and information (60) Dilution error (60) Wrong drug (60)	Yes (n = 5) Medication tray reorganization Medication cart top template Syringe labeling Infusion double check A medication practice guideline was developed	Single audit	NA
Mora-Capin et al^42^	Pediatric emergency department process	Treatment Diagnostic test Discharge management	Yes (n = 19) Development, implementation, and validation of a new, patented pediatric triage scale (TRIPED-GM) Simulation program to train multidisciplinary teamwork in the care of children with emergent conditions Assignment of roles in the care of critically ill patients (at the beginning of each shift) Electronic prescription protocols with automated dose calculation and application of maximum dose restrictions Urgent microbiological testing sample tracking and traceability record Safe transport protocol	New risk map (RM)	The level of risk decreased by 20% in the 2019 RM. To assess the impact of the 19 implemented IAs, we specifically analyzed the 46 prioritized risks (FMs) that these strategies were meant to address. Sixty percent of these FMs went from being classified as high-risk in 2017 to being classified as moderate risk in 2019.
Najar et al^43^	NICU drug prescription and administration	Errors in prescription method (60) Incomplete comment in physician order (40) Allergic reaction of the patient to the prescribed drug (40)	Yes (n = 13) Pharmacists’ participation on physical rounds, approval of all prescription medication by pharmacists Computerized physician order entry Disapproval of the incomplete comment in prescription by pharmacists and organizations Identification of all allergic reactions of a patient before admission or transfer to ward Recording of adverse reactions to drugs in patient’s medical history	NA	NA
Pino et al^44^	Unfractioned heparin (UFH) use	Mathematical errors (NA) Multiple practices are acceptable/requirements not known (NA) UFH can be given at any (incorrect) time (NA)	Yes (n = 43) Standardize required charting and documentation for all central lines via standard central line form that will cross encounters with ease of access Update and simplify central line flushing policy Create video to standardize central line care and flushing education Develop and implement easy access to patient-specific, cumulative UFH dose calculator Develop anticoagulation stewardship program	NA	The median RPN calculated for the process assuming implementation of the 22 countermeasures was 40, considered statistically different (*P* < 0.0001).
Webster et al^45^	Infant misidentification or abduction	Patients are escorted from the emergency department to the psychiatric unit through the circumcision area where infants may be lying (48) Safety and communication drills are only once a year (48) Aggressive individuals allowed to enter the labor and delivery ward due to propped doors, no posted security, or others holding the door open (48)	Yes (n = 4) Setting up a front desk near the main entrance and having a greeter stationed there A box of keepsake items would be made available to mothers whose infant had been taken into custody by the Child Protective Services (CPS) Infant abduction drills were carried out about once a year Each newborn is fitted with 3 wristbands and 1 security band, if medically appropriate	NA	NA
Williams et al^46^	Managing change-associated risks in pediatric trauma center	Equipment/supply availability (16) Staffing (16) System issues (issues related to process or procedures that do not work as well as anticipated in the clinical setting (16)	Yes (n = 18) New staffing policy: if someone calls out, backup RN required to come in to help immediately (previously home call) Trauma drug kit with additional emergency drugs prepositioned in the core OR and in the emergency department Anaesthesiologist/CRNA on call to carry trauma pager Rave text messages may be carrier dependent and therefore delayed Review of protocol and reeducation of blood bank staff Identify roles upon arrival/prebrief if time allows	Assessment of economic benefits	NA
Zhang et al^48^	Human milk process	NA	Yes (n = 15) A separate milk processing area A milk inventory system with bar-code Increase storage capacity by using larger fridge FTE designated to milk handling and prep Private patient rooms	NA	NA
Bagnasco et al^35^	Communication in emergency department	Filling in chart for transfer/discharge. Chart not shared (366) Drug administration (384) Report on admission on accident (512)	Yes (n = 3) Reorganizing the communication with communication training (SPAR) Training a multidisciplinary team to enhance the attitude to teamwork Standardise verbal and nonverbal communication through role-playing and simulation	NA	NA
Nguyen et al^50^	Nurse medication administration process	Drug dose prepared with inadequate technique (551) Drug dose inadequately identified (551) Absence of advice to the patient (551)	Yes (n = 22) Review of how materials are being arranged in medical trolleys and preparation areas Create a checklist to initialize drug deliveries Increase accessibility of contact sheets by adding them to the medical trolley binder Review the relevance of adverse reactions printed on the medication administration sheet. Add computer stations in targeted units to increase accessibility	NA	NA
Chandonnet et al^49^	Central line–associated bloodstream infections	Contamination (36) Suboptimal environment of care (24) Inadvertent disconnection dislodgement (27)	Yes (n = 23) Carry out expiration date checks, update the list of most commonly used drugs to be provided on the medical trolleys, audit drug returns Send all medicines in the patient’s possession to the pharmacy for checking and labeling on arrival of the patient Identify the drawer containing oral and intravenous supplies using labels Check how the materials are arranged in the medical trolleys and in the areas used to prepare the patient Preparation of an initialization checklist for medication deliveries	Ongoing audits	NA
Molavi-Taleghani et al^47^	Pediatric surgery process	Request for unnecessary tests by the physician (9) Error in entering the test results (9) The patient’s delay in entering the ward for making the organizations (9)	Yes (n = 18) Conducting regular evaluation and providing feedback to staff Reducing workload and addressing staff shortages Improving the patient identification process and revise guidelines for correct patient identification Conducting teamwork training Defining the written responsibilities for the admission unit and announcing them Physicians’ training on how to write the prescription	NA	NA

Ten studies presented evaluations of the proposed improvements’ impact. The evaluations had mainly been conducted by audits including ongoing audits,^49,50^ monthly audits,^37,40^ and periodical audits.^38,41^ Feedback had been collected using surveys.^34^ Economic benefits^46^ and the number and type of incident reports had been used as an evaluation method.^36^

New RPNs were recalculated and reported after corrective actions had been implemented.^34,38,40,42^ In one study, new RPNs were calculated for the process by assuming the implementation of countermeasures.^44^ Studies that aimed to reduce the risk of errors in prescribing and administering drugs^38^ and to design a safer approach to intravenous drug infusions^34^ found no high-risk RPNs after implementing interventions.

## DISCUSSION

This scoping review presents an overview of the use of different Failure Mode and Effect Analysis methods (FMEA, FMECA, and HFMEA) in pediatric and adolescent hospital care based on 18 studies showing their effectiveness in improving patient safety and quality.

Most risk assessments focused on medication processes, as adverse events (AEs) in pediatric care often involve medication and fluid management.^4–6^ FMEA methods helped identify potential medication-related AEs and implement improvement actions proactively. This is noteworthy in relation to the preventability of AEs.^6,7^

In addition, it is notable that certain FMEA methods were suitable for the assessment of very specific processes, for example, infant misidentification and abduction,^45^ which are unique events because the patients are minors.^1^ Process selection allows managers to make decisions based on various patient safety metrics, such as patient dissatisfaction^47^ or incident reports.^36^ Regardless of process type, it is beneficial to patient safety and quality to use a failure mode or a similar method to prevent AEs^7^ and to avoid serious consequences for patients^9^ and unnecessary costs for organizations.^10,11^

Pediatric hospital care typically offers a variety of specialized care. FMEA methods have been used in various pediatric health care settings.^19–21^ In the selected review studies, they had typically been used in the PICU^34,39,40^ and the NICU.^37,43,45,48,49^ In these kinds of care environments, medication, and fluid management are typical care interventions.^3–5^ However, studies of the use of FMEA methods in child psychiatric care were not found in this review, possibly because of the individuality of psychiatric care processes. Nevertheless, FMEA methods are also suitable for assessing a restricted part of a process, for example, communication.^35^

Unlike AE reporting,^7^ FMEA methods require a multidisciplinary team,^16^ but the team size can vary. Even a small, diverse team can perform effective risk assessment,^43,47^ which is important given health care staffing challenges. This is important information for health care organizations because FMEA methods are described as time-consuming.^37^ Instead of obtaining a certain number of participants, it is more important to have members from different professions. They can contribute different viewpoints on the topic under evaluation.^33^

Concrete results from the evaluation are obtained by identifying areas for improvement. Typically, improvement actions target high-risk events with the highest RPNs. For example, miscalculations (high RPN) can be avoided through double-checking and signing for each medicine prescription (improvement action).^36^ We concentrated on reporting high-risk failure modes. It is noteworthy to pay attention to FMs with low RPNs because they can cause additional work and lower the quality of care.

Care environments and care interventions change over time; thus, reassessment is essential to ensure quality improvement. RPNs had been recalculated and reported in some studies.^4,38,40,42^ A decrease in RPNs is one way to prove the effectiveness of these methods. This could motivate the participants to use similar methods in the future. Redesigning a process can create unintended adverse consequences that can be captured by redoing FMEA. At the same time, the awareness of potential safety risks increases among the participants.^33,37^

### Strengths

This scoping review is based on a systematic search technique. It was designed, tested and approved in collaboration with an information specialist. The criteria for inclusion and exclusion were clear, and the data extraction was comprehensive. This review is a summary of pediatric hospital care processes that have been analyzed with the FMEA methods, and it provides an opportunity to become familiar with these methods.

### Limitations

The purpose of the study selection process was to produce a coherent and analyzable set of data. It was challenging to compare the results of the FMEA analyses because each set of results had been reported according to the purpose of the respective study, and the method had been used to evaluate different processes. Failure Mode and Effects Analysis can be modified, which is an advantage for the user of the method but makes the comparability of outcomes difficult. The quality of the studies was not assessed in this review, but all the studies had been peer-reviewed.

## CONCLUSIONS

FMEA, FMECA, and HFMEA have been successfully used to ensure patient safety in pediatric and adolescent hospital care. They are effective methods for realizing possible failures in health care processes and can be used in quality improvement and risk reduction. However, considering how common AEs are, FMEA methods are not sufficiently used in work to improve patient safety or quality in pediatric hospital care. These methods should be tested for how they assess risks in processes other than medication. The experiences of those who have used these methods could be studied.
